# Yttrium-90-labeled microsphere tracking during liver selective internal radiotherapy by *bremsstrahlung *pinhole SPECT: feasibility study and evaluation in an abdominal phantom

**DOI:** 10.1186/2191-219X-1-32

**Published:** 2011-12-02

**Authors:** Stephan Walrand, Michel Hesse, Georges Demonceau, Stanislas Pauwels, François Jamar

**Affiliations:** 1 Center of Nuclear Medicine, Université Catholique de Louvain, Avenue Hippocrate 10, Brussels, 1200, Belgium; 2 Nuclear Medicine, Sint-Elisabeth Ziekenhuis, Zottegem, 9620, Belgium

**Keywords:** bremsstrahlung, pinhole, SPECT, SIRT, yttrium-90, microsphere, dosimetry

## Abstract

**Background:**

The purpose of the study is to evaluate whether a pinhole collimator is better adapted to *bremsstrahlung *single photon emission computed tomography [SPECT] than parallel-hole collimators and in the affirmative, to evaluate whether pinhole *bremsstrahlung *SPECT, including a simple model of the scatter inside the patient, could provide a fast dosimetry assessment in liver selective internal radiotherapy [SIRT].

**Materials and methods:**

*Bremsstrahlung *SPECT of an abdominal-shaped phantom including one cold and five hot spheres was performed using two long-bore parallel-hole collimators: a medium-energy general-purpose [MEGP] and a high-energy general-purpose [HEGP], and also using a medium-energy pinhole [MEPH] collimator. In addition, ten helical MEPH SPECTs (acquisition time 3.6 min) of a realistic liver-SIRT phantom were also acquired.

**Results:**

Without scatter correction for SPECT, MEPH SPECT provided a significantly better contrast recovery coefficient [CRC] than MEGP and HEGP SPECTs. The CRCs obtained with MEPH SPECT were still improved with the scatter correction and became comparable to those obtained with positron-emission tomography [PET] for the 36-, 30- (cold), 28-, and 24-mm-diameter spheres: CRC = 1.09, 0.59, 0.91, and 0.69, respectively, for SPECT and CRC = 1.07, 0.56, 0.84, and 0.63, respectively, for PET. However, MEPH SPECT gave the best CRC for the 19-mm-diameter sphere: CRC = 0.56 for SPECT and CRC = 0.01 for PET. The 3.6-min helical MEPH SPECT provided accurate and reproducible activity estimation for the liver-SIRT phantom: relative deviation = 10 ± 1%.

**Conclusion:**

*Bremsstrahlung *SPECT using a pinhole collimator provided a better CRC than those obtained with parallel-hole collimators. The different designs and the better attenuating material used for the collimation (tungsten instead of lead) explain this result. Further, the addition of an analytical modeling of the scattering inside the phantom resulted in an almost fully recovered contrast. This fills the gap between the performance of^90^Y-PET and *bremsstrahlung *pinhole SPECT which is a more affordable technique and could even be used during the catheterization procedure in order to optimize the^90^Y activity to inject.

## Background

A selective internal radiation therapy [SIRT] using^90^Y-labeled microspheres is a rapidly emerging treatment of unresectable, chemorefractory primary and metastatic liver tumors. The success of such therapeutic approach depends on (1) the expertise of the interventional radiologist to selectively catheterize the appropriate branch of artery, (2) the selection of patients with limited tumor burden, and (3) the determination of the maximal activity which can be safely injected to the patient. This determination is not achievable by angiography and is usually performed using empirical formulas, such as the partition model [1]. Pre-therapy single photon emission computed tomography [SPECT] using^99m^Tc-labeled macroaggregates [^99m^Tc-MAA] is mainly intended to rule out patients who display a liver-to-lung shunt in excess of 20% [1,2]. Even if^99m^Tc-MAA SPECT shows some usefulness in simulating the liver-SIRT procedure [3-5],^90^Y-microspheres differ from^99m^Tc-MAA by the higher number of particles injected during the therapeutic procedure, which could lead to a more pronounced embolic effect [6]. Imaging the actual^90^Y-microsphere deposition during the liver SIRT appears thus preferable.

Gupta et al. [7] showed the feasibility of iron-labeled microsphere tracking during transcatheter delivery in rabbit liver by magnetic resonance [MR] imaging. In this paper, cosigned by R. Salem, the authors concluded: 'Although quantitative *in vivo *estimation of microsphere biodistribution may prove technically challenging, the clinical effect could be enormous, thus permitting dose optimization to maximize tumor kill while limiting toxic effects on normal liver tissues.' However, human liver SIRT appears quite incompatible with MR: the X-ray angiographic imager will difficultly be implemented around the MR table, and the long duration of liver SIRT, which can take hours when the arterial tree is challenging, can unlikely be fitted into clinical MR agenda.

Several methods are already clinically used to assess the microsphere deposition after SIRT and check that the procedure has been performed as expected. Conventional *bremsstrahlung *imaging is already widely used in order to qualitatively assess biodistribution after^90^Y liver SIRT [8-17]. However, in the absence of a photopeak, SPECT imaging of^90^Y is dependent on the continuous *bremsstrahlung *X-rays. Although numerous correction methods have been proposed for parallel-hole collimator *bremsstrahlung *SPECT, the reached accuracy is still insufficient to safely determine the maximal activity to inject in each patient (see Walrand et al. [18] for an extensive review of the correction methods and applications).

More recently, the development of^90^Y-positron-emission tomography [PET] imaging [19-23] offers the unique opportunity to easily assess the actual absorbed dose delivered in^90^Y SIRT. Early human data have already provided a promising relationship between tumor dose and cell survival fraction [18,22]. However, the very low positron abundance (32 out of a million decays) required the use of long acquisition times (> 30 min).

To the best of our knowledge, *bremsstrahlung *SPECT using a pinhole collimator was never investigated for a human-directed application. This likely results from the fact that a pinhole collimator has a small field of view [FOV] and thus, for the imaging of large organs, results in lower SPECT performances compared with those obtained using parallel-hole collimators. However, in *bremsstrahlung *SPECT, the different designs (the pinhole collimator is almost an empty volume where high-energy X-rays cannot scatter down into the acquisition energy window) and the better attenuating material used for the collimation (tungsten rather than lead) could result in better *bremsstrahlung *SPECT performances using the pinhole collimator.

The purpose of the study is to evaluate whether a pinhole collimator is better adapted to *bremsstrahlung *SPECT than parallel-hole collimators and in the affirmative, to evaluate whether pinhole *bremsstrahlung *SPECT, including a simple previously published model of the scatter inside the patient [24,25], could provide a fast dosimetry assessment in liver SIRT. For comparison, a^90^Y time-of-flight [TOF]-PET acquisition was also acquired.

## Materials and methods

### Sphere phantom acquisitions

**Figure 1:**
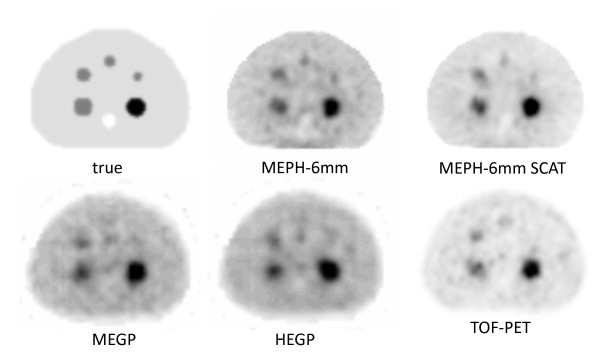
**Hot and cold sphere phantoms**. The figure shows transverse slices passing through the spheres' center for the different acquisitions with reconstructions of four iterations × eight subsets. Slices are shown for general information; the purpose of the study is for quantitative distribution assessment instead of diagnostic imaging. The true activity distribution is represented with the same voxel size than the reconstructions.

An abdominal-shaped container (31 × 23 cm^2 ^cross section × 8 cm length, 4.51 volume, Figure 1) was filled with 350 MBq of^90^Y (background + spheres). The container included six spheres with a diameter of 30, 36, 36, 28, 24, and 19 mm and a specific activity of 0, 7, 3.5, 3.5, 3.5, and 3.5 times that of the surrounding medium (background), respectively. A 30-min acquisition was performed on the GEMINI TF PET (Philips Medical Systems, Cleveland, OH, USA). One-hour acquisitions were performed on a single-head 400AC γ camera (1/2-in.-thick, 40-cm-diameter crystal, GE Healthcare, Haifa, Israel) in order to model a 30-min acquisition on a dual-head camera that is now the commercial standard. The acquisition energy window was limited from 50 to 150 keV in order to avoid the camera backscatter peak that is slightly above 150 keV [26]. Long-bore medium-energy general-purpose [MEGP] and high-energy general-purpose [HEGP] collimators (hole length 42 and 40 mm, septa thickness 1.4 and 3.2 mm, hole diameter 3.4 and 4.0 mm, respectively), and a medium-energy pinhole [MEPH] collimator (tungsten insert, aperture diameter 6 mm, focal length 26 cm, basal diameter 30 cm; the collimator was kindly provided by GE Healthcare) were investigated. Elliptical orbits were used to get the MEGP and HEGP collimators as close as possible to the phantom edge. For the MEPH collimator, the largest possible circular orbit was used in order to get the maximal transverse FOV.

### Collimator comparison

**Figure 2:**
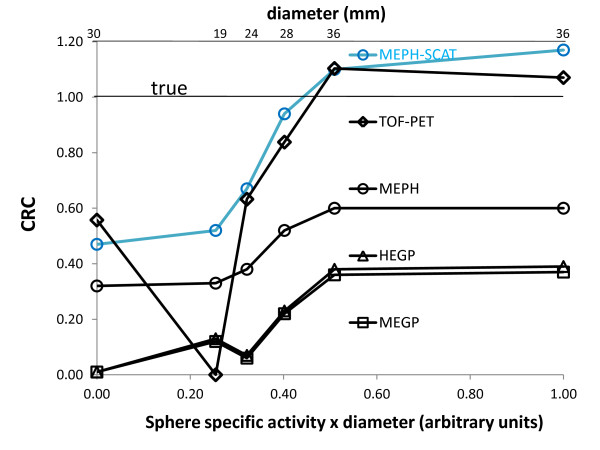
**Sphere CRC**. The figure shows the CRC as a function of the actual sphere specific activity times the sphere diameter with reconstructions of 20 iterations × 8 subsets. The true CRC is that obtained with the actual activity ratio.

**Figure 3:**
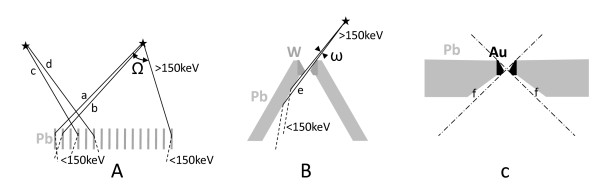
**Comparison of parallel-hole and pinhole collimator features**. The figure shows emission solid angles (Ω, *ω*) allowing a scattering down of the high-energy X-rays into the energy acquisition window. (**A**) In the parallel-hole collimator, note that Ω is the emission solid angle for the scatter paths (a) and also of the penetrating-scatter paths (b) that are reduced in the HEGP collimator compared to the MEGP one. These paths can also occur from the activity not geometrically seen by the crystal (c, d). (**B**) In the MEPH collimator used in the present study, scatter paths (e) mainly occur from the activity region that is not seen by the crystal. Due to the high attenuation and double conical shape of the tungsten insert (W), the emission solid angle for the penetrating-scatter path is too small to be drawn on the figure. (**C**) The optimized pinhole collimator for *bremsstrahlung *SPECT avoids these scattering paths (e) to prevent wall scattering of high-energy X-rays penetrating through the nose of the gold insert; an empty space (f) is left between the collimator housing and the extreme rays (dot-dash lines) passing through the aperture.

Contrast recovery coefficients [CRCs] obtained with the different collimators were compared on the sphere phantoms (Figure 2). All reconstructions were performed using ordered subset expectation maximization [OSEM] (eight subsets) up to 250 iterations. Despite the acquisition setup used, with the MEPH collimator, only a 20-cm-diameter centered circle could be imaged at all acquisition angles. To reduce distortion and loss of counts near the edges of the pinhole FOV and also to reduce the truncation artifact generated during the reconstruction, the voxels outside the phantom were set to zero in the initial estimate of the activity distribution. As this setting also slightly reduced the noise, the same was applied to the parallel-hole collimators as well (note that in a patient study, this region can be delineated from a coregistered computed tomography [CT] scan). The reconstruction voxel size was 4 mm for PET and 6.5 mm for SPECT. The TOF, attenuation, and scatter were accounted for in the PET reconstruction [27]. The path of the betas before X-ray emissions was taken into account: in the SPECT reconstruction iterations, the voxels were extended on each side by the beta mean range before projecting their activity. The geometrical point spread function [PSF] of the different collimators was also accounted for. For the pinhole SPECT, at 0° and 90°, the edge of the phantom was 2 cm close to the pinhole aperture. Due to the magnification, a voxel projected its activity on the crystal in a circle of 13-pixel diameter, i.e., on more than 100 pixels. Instead of using a multi-ray approach such as that proposed by Vanhove et al. [28], we developed a projector including an analytical approximation of the profile generated on the crystal by the geometrical projection of a voxel through the aperture. As the purpose was to purely compare the hardware performance, specific effects of *bremsstrahlung *resulting from the high-energy X-rays, such as collimator penetration-scattering and backscattering in the camera, were not corrected for, and an effective attenuation coefficient (*μ *= 0.13 cm^-1^) [29] was used in the geometrical projection in order to account for the scattering inside the phantom (Figure 3).

### Pinhole SPECT with scatter modeling

To assess the 'intrinsic' CRC that can be reached by pinhole SPECT, i.e., not corrupted by the physical effects occurring in the emission medium, the continuous energy X-ray scattering in the phantom was modeled using an adapted version of a previously proposed analytical model [24,25].

Contrary to^99m^Tc, with^90^Y, each point of the phantom received a continuous energy spectrum of rays coming from each source in the phantom. As a result, scattered X-rays having an energy ranging in the energy acquisition window can occur in all directions. This difference was approximated by assuming an isotropic scattering emission in the analytical scatter model (see Appendix 1). With this assumption, the projection with scatter modeling *P*_scat _of the activity estimate *A*^n ^is simply obtained by adding a spatially variant convolution of this estimate with an effective attenuation kernel followed by the geometrical projection $P_{\text{geom}}^{\mu }$:

(1) $$P_{\text{scat}}\left(A^{\text{n}}\left(\vec{x}\right)\right)=P_{\text{geom}}^{\text{μ}}\left(A^{\text{n}}\left(\vec{x}\right)+\alpha \;\rho \left(\vec{x}\right)\;∭\;d\vec{X}\;e^{-\oint_{}{}_{\vec{X}}^{\vec{x}}\overset{\wedge }{\mu }\left(\vec{y}\right)d\vec{y}}A^{\text{n}}\left(\vec{X}\right)\right),$$

where  is the linear integration of the effective attenuation coefficient $\overset{\wedge }{\mu }\left(\overset{\to }{y}\right)$ along the straight line from the point $\overset{\to }{X}$ to the scattering point $\overset{\to }{x}$, and $\rho \left(\overset{\to }{x}\right)$ is the density at the point $\overset{\to }{x}$ (zero in air). In liver SIRT, the attenuation is almost homogeneous, and the linear integration  in Equation 1 reduces to . Using fast Fourier transform, the additional convolution in Equation 1 did not increase the computation time per iteration.

The effective attenuation coefficient $\overset{\wedge }{\mu }$ was obtained by fitting the scatter profile along a tank filled with water and placed on a MEGP collimator, with a^90^Y point source placed on one side of the tank (see Appendix 1). The scatter fraction *α *was obtained from a pinhole SPECT of a 20-cm-diameter Perspex cylinder (Philips Medical Systems) centered in the FOV, filled with water and containing an off-centered^90^Y point source. The scatter fraction *α *was fitted to obtain the best agreement between the computed projections of this cylindrical phantom using Equation 1 and the measured planar views. As the scattering is now accounted for, the attenuation coefficient *μ *in Equation 1 is now the total attenuation coefficient and was set to the water attenuation coefficient at the middle of the energy acquisition window (*μ *= 0.17 cm^-1^), both in the scatter modeling procedure and in the phantom pinhole SPECT reconstruction. The projection used in the collimator comparison corresponds to Equation 1 with *α = 0 *and *μ *= 0.13 cm^-1^.

### Quantitative assessment

The performances of the collimators were evaluated using the CRC obtained for the spheres:

(2) $$\text{CRC = }\frac{C^{\text{meas}}-1}{C^{\text{true}}-1},$$

where *C*^meas ^and *C*^true ^are the measured and true spheres to background specific activity ratios, respectively. The measured specific activity of a sphere was the mean specific activity obtained in a spherical volume of interest [VOI] centered on the sphere and having the actual diameter of the sphere. The background specific activity was the mean specific activity in the phantom voxels outside these sphere VOIs. The CRC is equal to 1 for an ideal reconstruction for both cold and hot spheres.

### Liver-SIRT phantom acquisition

The same abdominal-shaped container filled with water was used as the scattering medium. A 5-cm-diameter cylinder filled with a K_2_HPO_4 _solution was set in the container in order to model the spine attenuation. A complex distribution activity pattern corresponding to a typical liver SIRT was modeled inside an 800-ml box set in the anatomical position of the right liver. In the right area of this 800-ml box, a necrotic heterogeneous tumor was modeled by a shell of five active 13-ml bottles (diameter 2.4 cm, length 2.8 cm) surrounding a cold core (a 13-ml bottle filled with water). In the left area, an isolated tumor was modeled by an active 13-ml bottle. The healthy right liver (709 ml) included four compartments: three active 58-ml bottles (one close to the shell and two close to the isolated tumor) and the 535-ml space in between and around the bottles. A total activity of 1.4 GBq was used. Activities of the different compartments are shown in Table 1.

**Table 1:** Abdominal phantom compartment activities assessed by the MEPH with scatter correction [MEPH-SCAT] SPECT

	True			3.6-min Acquisition time		1-min Acquisition time	
	Volume (ml)	RSA	% of 1.4 GBq	% of 1.4 GBq	RD (%)	% of 1.4 GBq	RD (%)
Core	13	0	0	1.14 ± 0.13	NA	1.20 ± 0.17	NA
Shell	52	4	27.31	20.79 ± 0.35	-24	20.42 ± 0.59	-25
Isolated tumor	13	4	5.46	4.34 ± 0.10	-21	4.32 ± 0.27	-21
Healthy liver 1	34	1	3.73	3.22 ± 0.15	-14	3.26 ± 0.18	-13
Healthy liver 2	58	0.25	1.60	2.46 ± 0.59	54	2.12 ± 0.20	32
Healthy liver 3	58	0.5	3.20	4.03 ± 0.26	26	4.11 ± 0.31	28
Healthy liver 4	58	0.5	3.20	4.25 ± 0.59	33	4.33 ± 0.40	35
Total healthy liver	709	NA	67.23	73.73 ± 0.41	10	74.06 ± 0.57	10

Relative specific activities (RSA; healthy liver is set to 1) and percentage of total activity (mean ± SD) in the liver-SIRT phantom for the different regions: necrotic tumor (core and shell), isolated tumor, and healthy liver regions (1: VOI sample far from the tumors; 2, 3, 4: cylinders; total healthy liver: the whole region beside the tumors). RD, relative deviation; NA, not applicable.

**Figure 4:**
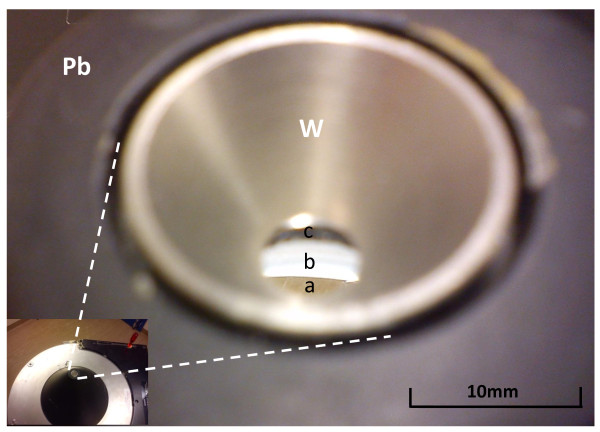
**MEPH collimator**. The figure shows a view of the MEPH aperture collimator (from top to bottom in Figure 3B with an angle of 45°) set on the carrier trolley. Pb is the lead housing facing the targeted activity. W is the tungsten insert. (a) The floor of the room. (b) The inner side of the conical lead housing. (c) The bottom part of the lead thread in which the insert is screwed.

**Figure 5:**
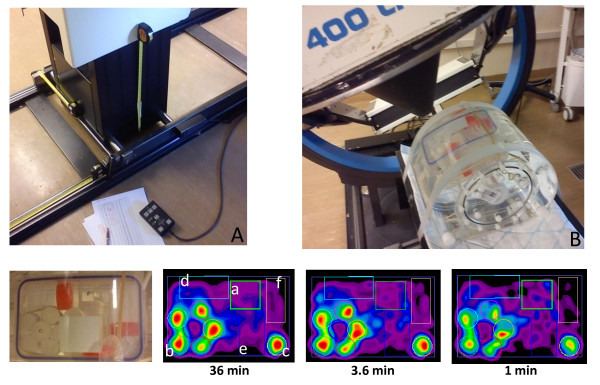
**Acquisition and reconstruction of the abdominal phantom modeling hepatic metastases**. The figure shows liver-SIRT phantom acquisition and reconstruction. (**A**) The bed holder and the three tape measures. (**B**) The counterweight lever system that does not allow pure radial motion. The bottom row shows the images of the liver model and reconstructed oblique slices passing through the middle of the liver model for 36-, 3.6-, and 1-min acquisitions. (a) The VOI sample in the healthy liver. (b) The necrotic tumor and (c) the isolated tumor with both specific activities fourfold that of the healthy liver. (d, e, f) The cylinders with specific activities 0.5, 0.5, and 0.25 times that of the healthy liver, respectively. The cylinder (f) section is smaller than that of cylinders (e) and (d) because cylinder (f) was not centered.

A helical MEPH SPECT (two half rotations from -135° to 45° with a longitudinal pitch of 5.4 cm per half rotation; Figure 4) was manually performed on the GE 400AC camera in the following way. Three tape measures were fixed on the bed in order to note its position in the three directions (the camera does not allow radial motion for the detector, Figure 5). For 72 times, the camera was rotated by 5° and the bed shifted by 1.5 mm in the longitudinal direction manually. At each angle, (1) the bed was vertically and horizontally shifted in order to keep at least 10 cm between the pinhole aperture and the 800-ml box in order to avoid truncation artifacts; (2) the bed position in the three directions was reported; and (3) 10 frames of 3 s were recorded (matrix 128 × 128) by putting together the frame number *i *(*i *= 1,...,10) of all rotation angles, and 10 helical SPECTs of a 3.6-min acquisition time were generated. Due to all the manipulations, the total acquisition times was 3.5 h, so about 3 h just for the manual motions and the initialization of the dynamic acquisitions at each angle, making a trial on patients using this SPECT system impossible.

The 3.6-min helical SPECTs were reconstructed with OSEM (70 iterations × 8 subsets) including the analytical scatter model. The tumor and liver VOIs were drawn on a CT scan of the phantom, and the position of the set of VOIs was afterward tuned on the SPECT images (Figure 5). In liver SIRT, it can be approximated that the whole injected activity indefinitely remains in the liver and lungs and thus can be entirely imaged. As a result, the percentage of activity taken up by the different compartments was obtained by computing the ratio of the counts in the compartment VOI with the total count in the image. After time integration of the physical decay and summation-multiplication by the S factors between the different compartments, this determines the tissue dosimetry expressed in milligrays per megabecquerel [mGy/MBq] [30]. These S factors can be computed for each target ← source compartment by convolving a three-dimensional [3-D] mask of the source compartment VOI with a dose deposition kernel [31]. After analyzing the data, it was noted that the reproducibility of the 3.6-min acquisition time helical pinhole SPECTs was sufficiently good to expect useable results using shorter acquisition times, so we decided to generate pseudo 1-min helical SPECTs by keeping only the odd pixel in the two directions of the acquisition matrix (one pixel on four).

## Results

### Collimator comparison

The central transverse slices obtained using the different systems showed that TOF-PET provided the best contrast for the 36-, 30-, 28-, and 24-mm-diameter spheres, while MEPH-6-mm SPECT provided the best CRC for the 19-mm-diameter sphere (Figure 1). This was confirmed in Table 2 and Figure 2, showing the quantitative CRC obtained by the different systems for all spheres. For the cold and 28-mm hot spheres, the MEPH provided a CRC twice higher than that provided by the parallel-hole collimators and made the two smallest hot spheres clearly visible. The cold sphere CRC was also significantly improved.

**Table 2:** CRC of the hot and cold sphere phantoms

Diameter (mm) (act sph/bg)	30 (0)	19 (3.5)	24 (3.5)	28 (3.5)	36 (3.5)	36 (7)
TOF-PET^a^	0.56	0.01	0.63	0.84	1.10	1.07
MEPH-SCAT SPECT^a^	0.59	0.56	0.69	0.91	1.03	1.09
MEPH-SPECT^a^	0.32	0.33	0.39	0.52	0.60	0.60
HEGP-SPECT^a^	0.01	0.13	0.06	0.23	0.38	0.39
MEGP-SPECT^a^	0.01	0.12	0.06	0.22	0.36	0.37

^a^CRC of the different spheres obtained for reconstructions with 20 iterations × 8 subsets, except for the MEPH-SCAT shown for reconstructions with 70 iterations × 8 subsets. The iteration numbers are optimal for the hot spheres, but not for the cold ones, the CRC of which continues to slowly improve with the iteration number (see Appendix 2 for the convergence rate). According to Equation 2, an ideal reconstruction gives a CRC equal to 1 for both cold and hot spheres. TOF-PET, time-of-flight positron-emission tomography; MEPH-SCAT SPECT, medium-energy pinhole with scatter correction single photon emission computed tomography; MEPH-SPECT, medium-energy pinhole single photon emission computed tomography; HEGP-SPECT, high-energy general-purpose single photon emission computed tomography; MEGP-SPECT, medium-energy general-purpose single photon emission computed tomography; act sph/bg, ratio between the specific activity of the sphere and of the background.

### Pinhole SPECT with scatter modeling

The values obtained for the scattering modeling in Equation 1 were *α *= 1.97 × 10^-4 ^and $\overset{\wedge }{\mu }$ = 0.0697 cm^-1^. Using the scattering analytical model, MEPH provided similar results as those of the TOF-PET (Table 2), but with the need to perform significantly more iterations (see Appendix 2 about the CRC convergence rate). Table 1 and Figure 5 show the results obtained with the helical MEPH SPECT for the liver-SIRT phantom reconstructed with 70 iterations (eight subsets).

## Discussion

This study demonstrates the better hardware properties of a pinhole collimation (MEPH) for *bremsstrahlung *SPECT imaging. Further, the adaptation of a previously described analytical modeling of the scattering inside the patient leads to contrast recovery very close to those obtained with^90^Y-PET.

The better CRC obtained by MEPH compared with MEGP or HEGP collimators resulted from the reduced high-energy X-ray penetration in the tungsten insert of the pinhole compared to that of the lead septa of the parallel-hole collimators. Also, the pinhole collimator is almost an empty volume reducing the amount of high-energy X-rays scattering down into the acquisition energy window (Figure 3B) compared to parallel-hole collimators (Figure 3A). These features made the improvement especially noticeable for the cold sphere and the three smallest hot spheres (Table 2, Figure 2).

Using a simple analytical scatter model in the phantom, MEPH SPECT provides similar results than those of TOF-PET (Table 2, Figure 2), although TOF-PET is free of these collimator penetration-scatter and also of camera backscatter drawbacks. The results are even better for the smallest sphere that is hampered by the higher noise obtained in PET reconstruction as shown in Figure 1.

The analytical modeling of the scatter was derived from phantoms having different geometries, sizes, and distribution activities than those of the spheres and of the liver-SIRT phantom. This assures that the model can be applied to various patient corpulences. Also, the fact that the cold sphere CRC at the end converged to the same value than that of the hot sphere having the same diameter (see Appendix 2) proved that the background activity is well reproduced and that the analytical model does not underestimate or overestimate the scatter contribution. Furthermore, this model does not increase the computation time per iteration. Nevertheless, as the goal is to determine which maximal activity is still safe for the liver during the liver SIRT within a few minutes, it is of prime importance to further validate in patients the proposed method before its utilization in optimizing the injected activity. This validation could be performed by comparing the results with those obtained using a long-acquisition time PET (preferably TOF-PET) soon performed after the radioembolization.

The pinhole collimator used in our study was not designed for *bremsstrahlung *SPECT, and several features can still be improved. A gold or iridium insert and thicker pinhole lead walls can still reduce the contamination due to the penetration of the high-energy X-rays. The design of the collimator housing itself can be improved. Indeed, in conical housing pinhole, there is a possibility for the high-energy X-rays to pass through the aperture or through the nose of the aperture and then, to scatter on the pinhole inner lead walls down to an energy inside the acquisition energy window (Figure 3B, 4). Contrary to the parallel-hole collimator, these scatterings mainly occur from X-rays emitted in areas not geometrically seen by the crystal. Making the collimator housing cylindrical rather than conical, the insert will be inside a thick lead plate parallel to the crystal, and the scattering by the inner wall will be removed (Figure 3C). This housing shape will also have the benefit of removing the risk of hurting the patient.

Besides the optimization for *bremsstrahlung *imaging, the pinhole collimator should also be optimized to large-organ SPECT. This can be done by decreasing the focal length in order to increase the transverse FOV at a short distance to the aperture using the whole crystal surface (the MEPH collimator of the present study used only three-fourths of the crystal diameter). Multiple pinhole collimators should also be better adapted. Lastly, the aperture size and energy window should be optimized in relation with collimator effects modeling in the reconstruction process.

However, even with this suboptimal pinhole collimator, the results obtained for the liver-SIRT phantom showed that a 3.6-min helical MEPH SPECT with 70 iterations (eight subsets) is sufficient to obtain an accurate (relative deviation 10%) and reproducible (standard deviation [SD]/mean < 1%) estimation of the healthy liver activity that determines the maximal safe activity which can be injected (Table 1). The percentage of uptake in the different compartments was estimated versus the whole activity measured in the reconstruction. Thus, the computation of the compartment absorbed doses will require an accurate measure of the total delivered activity. Especially, the catheter and microsphere vial will have to be imaged or counted after the radioembolization.

Rather than to estimate the mean liver absorbed dose by multiplying the percentage taken up by the liver region reached by the microspheres with the S factor of this region, a voxel-based dosimetry could be obtained by convolving the reconstructed^90^Y distribution with a dose deposition kernel [18,20]. This will allow computation of the normal tissue complication probability using the equivalent uniform dose in order to take into account the liver irradiation heterogeneity. This can be done using Niemerko's model [32] and the normal tissue tolerance determined by Emami et al. [33]. The software performing this computation is already available [34], and recently, an improvement of Niemerko's model was proposed [35].

Using four commercial 4 × 8-core Xeon (Intel Corporation, Santa Clara, CA, USA) or 4 × 12-core Opteron (AMD, Sunnyvale, CA, USA) computers in a cluster, accurate results could be obtained in a 30-s computation time (see Appendix 2). The results obtained with the pseudo 1-min-helical acquisition (Table 2) supports that using an optimal pinhole collimator, it could be possible to reduce the acquisition time to 1 min. Although the small SDs obtained show that the statistic is sufficient, the reconstructed image is corrupted by more artifacts than for the sphere phantom where all the spheres were just in front of the collimator aperture. This likely resulted from the high pitch used (5.4 cm per half rotation). Ideally, the pitch should not be larger than the targeted final resolution (1 cm), requiring an acquisition software allowing automated helical SPECT that is not yet available on a commercial camera.

**Figure 6:**
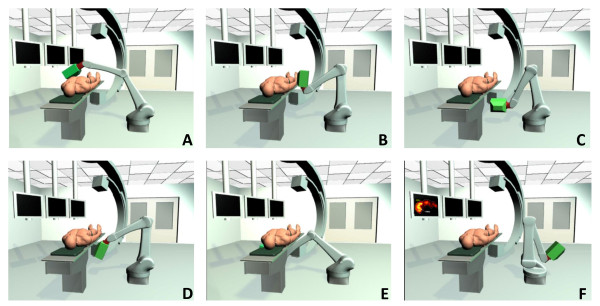
**Example of a multi-pinhole SPECT implementation in a catheterization room using a six-axis arm robot**. (**A**, **B**) The orbit motion above the patient. (**C**) The end-stage rotation drive of the robot rotates the detector around itself to turn the collimator side upward. (**D**, **E**) The orbit motion under the patient. (**F**) In home position, the system leaves the space around the patient free. During the orbit motion, the robot rotates slowly on its pedestal to provide a helical acquisition. See animation in Additional file 1.

Besides being more affordable than PET, the possibility to estimate the mean absorbed dose delivered to the healthy liver reached by microspheres in a few minutes by pinhole SPECT also offers new possibilities. Indeed, the price of a single-head gamma camera is only about tenfold that of a liver-SIRT procedure, and it could be advisable to install one in the catheterization room. The helical acquisition orbit could be performed using a six-axis industrial arm robot; in home position, the system will leave the space around the catheterization table free (Figure 6, see Additional file 1). These industrial robots [36] are very accurate (0.06 mm), can handle payloads up to 1 ton, are reasonably cheap (a 300-kg payload model costs about two liver-SIRT procedures), and their combined use with a gamma camera requires only to synchronize together the starts of the camera acquisition and of the robot motion. Such robots are already used in radiation therapy [37] or assisted surgery. State-of-the-art informatics driving systems are reliable and efficiently prevent any hurt to the patient.

## Conclusion

The use of pinhole SPECT reduces the disturbing interactions of the high-energy X-rays with the collimator. This would allow implementing a dosimetry assessment during the liver-SIRT procedure without displacing the catheter and at the end, injecting the optimal activity that provides the highest absorbed dose to the tumors still safe to the liver. This may definitely improve the patient outcome.

## Competing interests

The authors declare that they have no competing interests.

## Authors' contributions

SW conceived the method. SW, FJ, and GD participated in the design of the study. SW and MH developed the reconstruction algorithm. SW, MH, SP, and FJ participated in the data analysis and in the writing of the manuscript. All authors read and approved the final manuscript.

## Appendix 1

### Scatter model

The angular distribution *σ *(*θ*) of photon scattering is given by the Klein-Nishina formula [38]:

(3) $$\sigma \left(\theta \right)=\frac{r_{0}^{2}}{2}{\left(\frac{E}{E_{0}}\right)}^{2}\left(\frac{E_{0}}{E}+\frac{E}{E_{0}}-\sin^{2}\theta \right),$$

where *E *is the energy of the scattered photon, *E*_0 _is the initial energy of the photon, and *r*_0 _is the classical radius of the electron. *E*_0_, *E*, and *θ *are linked together by the Compton formula [38]:

(4) $$\frac{1}{E}-\frac{1}{E_{0}}=\frac{1-\cos\theta }{511},$$

where 511 (keV) is the energy of the electron at rest.

**Figure 7:**
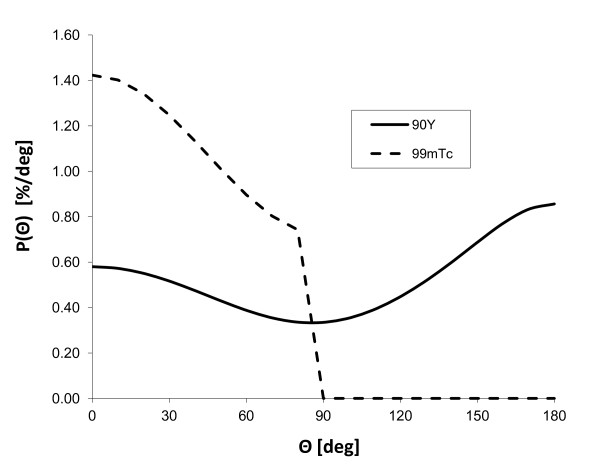
**Scattering angular distribution**. The figure shows the angular distribution *P*(*θ*) of single scatterings of a primary ray that drops its energy in the window (115, 140 keV) and (50, 150 keV) for^99m^Tc and^90^Y, respectively. The incident beam hardening is neglected (scatter and primary emission point close together).

For^99m^Tc (*E*_0 _= 140 keV), the Compton formula (Equation 4) shows that scattering angles higher than 80° in the phantom, or the patient, drop the gamma ray energy below the energy acquisition window. The angular distribution of the scattered photon detectable by the camera is thus given by the Klein-Nishina formula truncated above 80° (Figure 7).

The angular distribution *P*(*θ*) of single scatterings of a primary *bremsstrahlung *X-ray beam coming from a^90^Y source that drops its energy into the window (50, 150 keV) is:

(5) $$P\left(\theta \right)=\int_{50}^{150}\sigma \left(\theta \right)\;S\left(\frac{511}{511-E\left(1-\cos\theta \right)}E\right)dE,$$

where *S(E*_0_*) *is the *bremsstrahlung *X-ray yield at energy *E*_0 _reaching the scattering point; note that due to the attenuation, there is a hardening of the X-ray beam when the distance between the emission and scattering points increases. Due to the continuous energy spectrum up to 2.27 MeV of the^90^Y *bremsstrahlung *X-rays, all the scattering domain (0°, 180°) × (50, 150 keV) is targeted. The computation of Equation 5 using *S(E*_0_*) *obtained from Monte Carlo simulations [22] is given in Figure 7 and shows that contrary to^99m^Tc, the first scattering emission can be reasonably considered as isotropic for^90^Y. Successive scatterings will not fundamentally change this feature. As a result, while the high-energy continuous spectrum of^90^Y *bremsstrahlung *X-rays increases the contamination level of the scattering compared to^99m^Tc, it also simplifies the analytical model to approximate the scattering in the patient and its implementation in the iterative reconstruction that is now a simple additional convolution term.

### Effective attenuation coefficient fitting

**Figure 8:**
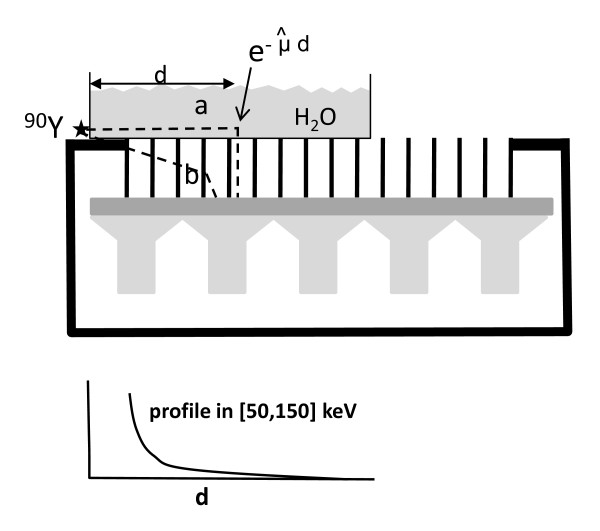
**Effective attenuation coefficient assessment**. The figure shows the experimental setup for the determination of the effective attenuation coefficient $\overset{\wedge }{\mu }$. The profile in the energy window (50, 150 keV) recorded on the camera equipped with a MEGP collimator is the result of two kinds of X-ray paths: (a) 90° scattering in the water and (b) penetration through the camera shielding and collimator septa.

**Figure 9:**
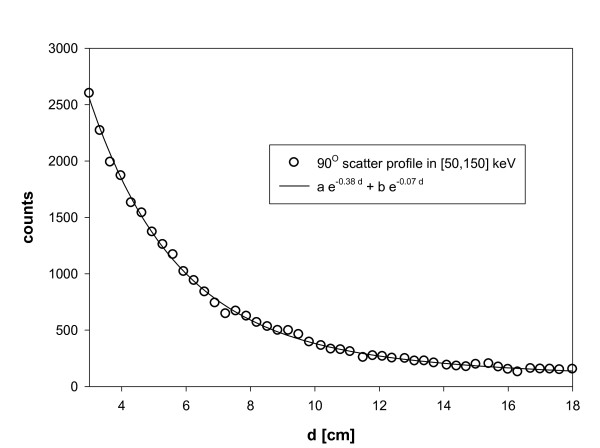
**Scatter profile**. The figure shows the fit of the scatter profile obtained from the setup shown in Figure 8. The fast exponential (0.38 cm^-1^) corresponds to X-ray penetration-scattering through the camera shielding and collimator septa.

The effective attenuation coefficient $\overset{\wedge }{\mu }$ was obtained by fitting the scatter profile along a tank filled with water and placed on a MEGP collimator with a^90^Y point source placed on one side of the tank (Figure 8). The profile corresponds to a 90° scattering which is in the middle of the scattering angle range possible in the phantom. The fit of the profile by a double exponential gave 0.0697 and 0.378 cm^-1 ^for the two exponent coefficients (Figure 9). The fast exponential decrease is due to the X-ray penetration-scattering through the camera shielding and collimator septa (Figure 8, path b); indeed, this attenuation coefficient is too high to be produced by water.

## Appendix 2

### Convergence rate

**Figure 10:**
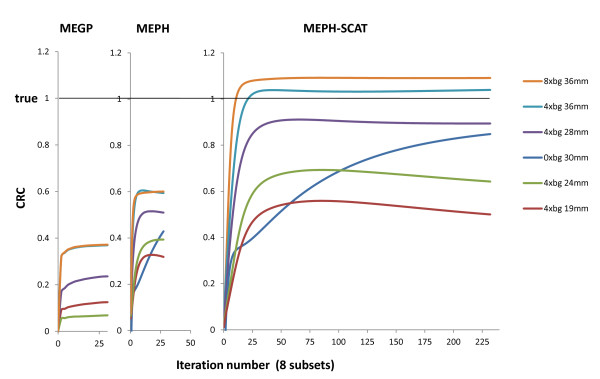
**CRC convergence rate per iteration of the spheres**. The figure shows iterations using eight subsets for the MEGP collimator and for the MEPH collimator with and without scattering correction. HEGP provided similar convergence rate than MEGP (data not shown). Note the presence of two phases in the convergence rate for the cold sphere.

Figure 10 shows the CRC convergence rate per iteration for the SPECT reconstructions. The inclusion of the scattering into the iteration significantly slows down the convergence rate. The reason is that the additional scattering contribution smoothes the back projection that appears in the multiplicative factor applied to the distribution estimate to obtain the next one. To overcome this drawback, some authors proposed to include smoothing factors, such as collimator PSF or scattering, only in the projection step [39]. However, this method does not longer exactly account for the Poisson nature of the noise and in this study, slightly degrades the results (data not shown). Figure 10 shows that the cold sphere CRC is null for the parallel-hole collimators and that for the pinhole collimator, a fast, but short, CRC increasing phase is prolonged by a slow growth up to a value near that of the hot sphere of a similar diameter. As the purpose in liver-SIRT dosimetry is mainly to study the partition of the injected activity between taking up healthy liver and taking up tumors, using eight subsets, it is not needed to go further than 70 and 20 iterations with and without using scatter modeling, respectively. The computation time on a 2 × 4-core Xeon 5335 server (core without hyperthreading available, MMX registers used) was 23 s per iteration for the pinhole SPECT, with or without scattering correction (6.5 mm voxel size on edge). A state-of-the-art current server offers up to 4 × 8-core Xeon 7540 with hyperthreading (price ≈ 25 k€), dropping down the iteration time to 3 s. Four of such servers in a cluster should provide the 70 required iterations in less than 1 min.

## Supplementary Material

Additional file 1 **SPECT animation**. An example of a multi-pinhole SPECT implementation in a catheterization room using a six-axis arm robot. Click here for file

## References

[B1] Kennedy A Nag S Salem R Murthy R McEwan AJ Nutting C Benson A Espat J Bilbao JI Sharma RA Thomas JP Coldwell D Recommendations for radioembolization of hepatic malignancies using yttrium-90 microsphere brachytherapy: a consensus panel report from the radioembolization brachytherapy oncology consortium Int J Radiat Oncol Biol Phys 2007 68 13 23 10.1016/j.ijrobp.2006.11.060 17448867

[B2] Lambert B Mertens J Sturm EJ Stienaers S Defreyne L D'Asseler Y 99mTc-labelled macroaggregated albumin (MAA) scintigraphy for planning treatment with 90Y microspheres Eur J Nucl Med Mol Imaging 2010 37 2328 2333 10.1007/s00259-010-1566-2 20683591

[B3] Ahmadzadehfar H Sabet A Biermann K Muckle M Brockmann H Kuhl C Wilhelm K Biersack HJ Ezziddin S The significance of 99mTc-MAA SPECT/CT liver perfusion imaging in treatment planning for 90Y-microsphere selective internal radiation treatment J Nucl Med 2010 51 1206 1212 10.2967/jnumed.109.074559 20660379

[B4] Garin E Rolland Y Lenoir L Pracht M Mesbah H Porée P Laffont S Clement B Raoul JL Boucher E Utility of quantitative Tc-MAA SPECT/CT for yttrium-labelled microsphere treatment planning: calculating vascularized hepatic volume and dosimetric approach Int J Mol Imaging 2011 2011 398051 21822489 10.1155/2011/398051 PMC3147134

[B5] Flamen P Vanderlinden B Delatte P Ghanem G Ameye L Van Den Eynde M Hendlisz A Multimodality imaging can predict the metabolic response of unresectable colorectal liver metastases to radioembolization therapy with yttrium-90 labeled resin microspheres Phys Med Biol 2008 53 6591 5603 10.1088/0031-9155/53/22/019 18978442

[B6] Bilbao JI Reiser MF Liver Radioembolization with 90Y Microspheres 2008 Berlin Heidelberg: Springer-Verlag

[B7] Gupta T Virmani S Neidt TM Szolc-Kowalska B Sato KT Ryu RK Lewandowski RJ Gates VL Woloschak GE Salem R Omary RA Larson AC MR tracking of iron-labeled glass radioembolization microsphres during transcatheter delivery to rabbit VX2 livers tumors: feasibility study Radiology 2008 249 845 854 10.1148/radiol.2491072027 18840788 PMC6944075

[B8] Sebastian AJ Szyszko T Al-Nahhas A Nijran K Tait NP Evaluation of hepatic angiography procedures and bremsstrahlung imaging in selective internal radiation therapy: a two-year single-center experience Cardiovasc Intervent Radiol 2008 31 643 649 10.1007/s00270-008-9298-4 18273668

[B9] Mansberg R Sorensen N Mansberg V Van der Wall H Yttrium 90 bremsstrahlung SPECT/CT scan demonstrating areas of tracer/tumour uptake Eur J Nucl Med Mol Imaging 2007 34 1887 10.1007/s00259-007-0536-9 17846767

[B10] Machac J Weintraub J Nowakowski F Mobley D Zhang Z Warner R Variations in liver perfusion patterns in patients with liver tumors undergoing therapy with yttrium-90 microspheres, studied with SPECT/CT J Nucl Med 2007 48 396P

[B11] Knesaurek K Muzinic M Zhang Z DaCosta M Machac J Comparison of visual and computer calculated coregistration of Y-90 and Tc-99m MAA SPECT/CT images in treatment of liver cancer J Nucl Med 2008 49 112P18077536

[B12] Knesaurek K Machac J Muzinic M DaCosta M Zhang Z Heiba S Quantitative comparison of yttrium-90 (90Y)-microspheres and technetium-99m (99mTc)-macroaggregated albumin SPECT images for planning 90Y therapy of liver cancer Technol Cancer Res Treat 2010 9 253 262 20441235 10.1177/153303461000900304

[B13] Moore S Park M Mueller S Activity estimation performance in Y-90 microsphere bremsstrahlung SPECT J Nucl Med 2009 50 1433

[B14] Tehranipour N AL-Nahhas A Canelo R Stamp G Woo K Tait P Gishen P Concordant F-18 FDG PET and Y-90 bremsstrahlung scans depict selective delivery of Y-90-microspheres to liver tumors: confirmation with histopathology Clin Nucl Med 2007 32 371 374 10.1097/01.rlu.0000259568.54976.bd 17452865

[B15] Simon N Feitelberg S Scanning bremsstrahlung of yttrium-90 microspheres injected intra-arterially Radiology 1967 88 719 724 6020936 10.1148/88.4.719

[B16] Gnanasegaran G Buscombe JR O'Rourke E Caplin ME Purfield D Hilson AJW Bremsstrahlung imaging after intra-arterial 90Y lanreotide radionuclide therapy for carcinoid liver metastases Nucl Med Commun 2005 26 284 285

[B17] Luo J Rao P Zimmer M Polis M Mistretta M Spies S Imaging technique in estimating lung shunting of yttrium-90 microspheres Med Phys 2005 32 1913

[B18] Walrand S Flux GD Konijnenberg MW Valkema R Krenning EP Lhommel R Pauwels S Jamar F Dosimetry of yttrium-labeled radiopharmaceutical for internal therapy: yttrium-86 or -90 imaging? Eur J Nucl Med Mol Imaging 2011 10.1007/s00259-011-1771-7 21484382

[B19] Lhommel R Goffette P Van den Eynde M Jamar F Pauwels S Bilbao JI Walrand S Yttrium-90 TOF PET scan demonstrates high-resolution biodistribution after liver SIRT Eur J Nucl Med Mol Imaging 2009 10.1007/s00259-009-1210-1 19618182

[B20] Lhommel R van Elmbt L Goffette P Van den Eynde M Jamar F Pauwels S Walrand S Feasibility of yttrium-90 TOF-PET based dosimetry in liver metastasis therapy using SIR-spheres Eur J Nucl Med Mol Imaging 2010 10.1007/s00259-010-1470-9 20422185

[B21] Werner MK Brechtel K Beyer T Dittmann K Pfannenberg C Kupferschläger J PET/CT for the assessment and quantification of 90Y biodistribution after selective internal radiotherapy (SIRT) of liver metastases Eur J Nucl Med Mol Imaging 2009 10.1007/s00259-009-1317-4 19997914

[B22] Lhommel R Walrand S van Elmbt L Pauwels S Jamar F Dose-response relationship in liver-SIRT: Y90 TOF-PET versus Tc99m-MAA SPECT based dosimetry Eur J Nucl Med Mol Imaging 2010 37 S201 10.1007/s00259-009-1319-2 20422185

[B23] Gates VL Esmail AAH Marshall K Spies S Salem R Internal pair production of 90Y permits hepatic localization of microspheres using routine pet: proof of concept J Nucl Med 2010 52 72 76 21149493 10.2967/jnumed.110.080986

[B24] Walrand SH van Elmbt LR Pauwels S Quantitation in SPECT using an effective model of the scattering Phys Med Biol 1994 39 719 734 10.1088/0031-9155/39/4/005 15552080

[B25] Cao ZJ Frey EC Tsui BMW A scatter model for parallel and converging beam SPECT based on the Klein-Nishina formula IEEE Trans Nucl Sci 1994 41 1594 1600 10.1109/23.322954

[B26] Rault E Staelens S Van Holen R De Beenhouwer J Vandenberghe S Accurate Monte Carlo modelling of the back compartments of SPECT cameras Phys Med Biol 2011 56 87 104 10.1088/0031-9155/56/1/006 21119230

[B27] Surti S Kuhn A Werner ME Perkins AE Kolthammer J Karp JS Performance of Philips Gemini TF PET/CT scanner with special consideration for its time-of-flight imaging capabilities J Nucl Med 2007 48 471 480 17332626

[B28] Vanhove C Andreyev A Defrise M Nuyts J Bossuyt A Resolution recovery in pinhole SPECT based on multi-ray projections: a phantom study Eur J Nucl Med Mol Imaging 2007 34 170 180 10.1007/s00259-006-0225-0 16953400

[B29] Shen S DeNardo GL DeNardo SJ Quantitative bremsstrahlung imaging of yttrium-90 using a Wiener filter Med Phys 1994 21 1409 1417 10.1118/1.597198 7838052

[B30] Stabin MG Fundamentals of Nuclear Medicine Dosimetry 2008 New York: Springer

[B31] Walrand S Jamar F van Elmbt L Lhommel R Bekonde EB Pauwels S 4-Step renal dosimetry dependent on cortex geometry applied to 90Y peptide receptor radiotherapy: evaluation using a fillable kidney phantom imaged by 90Y PET J Nucl Med 2010 51 1969 1973 10.2967/jnumed.110.080093 21078802

[B32] Niemierko A William Hendee: AAPM A unified model of tissue response to radiation Proceedings of the 41st AAPM Annual Meeting: July 25-29 1999; Nashville, Tennessee 1999 1100

[B33] Emami B Lyman J Brown A Coia L Goitein M Munzenrider JE Shank B Solin LJ Wesson M Tolerance of normal tissue to therapeutic irradiation Int J Radiat Oncol Biol Phys 1991 21 109 122 2032882 10.1016/0360-3016(91)90171-y

[B34] Gay HA Niemierko A A free program for calculating EUD-based NTCP and TCP in external beam radiotherapy Physica Medica 2007 23 115 125 10.1016/j.ejmp.2007.07.001 17825595

[B35] Luxton G Keall PJ King CR A new formula for normal tissue complication probability (NTCP) as a function of equivalent uniform dose (EUD) Phys Med Biol 2008 53 23 36 10.1088/0031-9155/53/1/002 18182685

[B36] KUKA http://www.kuka-robotics.com/en/products/industrial_robots

[B37] Kraus-Tiefenbacher U Scheda A Steil V Hermann B Kehrer T Bauer L Melchert F Wenz F Intraoperative radiotherapy (IORT) for breast cancer using the intrabeam™ system Tumori 2005 91 339 345 16277101 10.1177/030089160509100411

[B38] Bjorken JD Drell SD Relativistic Quantum Mechanics 1964 New York: McGraw-Hill Inc.

[B39] Kamphuis C Beekman FJ van Rijk PP Viergever MA Dual matrix ordered subsets reconstruction for accelerated 3D scatter compensation in single-photon emission tomography Eur J Nucl Med 1997 25 8 18 10.1007/s002590050188 9396869

